# Speech characteristics and their association with neuroimaging and CSF biomarkers of AD pathology

**DOI:** 10.1002/alz.089486

**Published:** 2025-01-09

**Authors:** William Simpson, Michael J Spilka, Mengdan Xu, Jessica Robin

**Affiliations:** ^1^ Winterlight Labs, Toronto, ON Canada; ^2^ Winterlight Labs (Cambridge Cognition), Toronto, ON Canada

## Abstract

**Background:**

Digital speech assessment shows promise as an objective and low burden measure of progressive speech and language changes in AD. While associations with clinical endpoints are well established, less is known about how computational speech features align with biomarkers of AD pathology. The current analysis sought to examine these associations both cross‐sectionally and longitudinally.

**Method:**

Speech samples were collected during a Phase 2/3 clinical trial in mild‐to‐moderate AD. Participants completed clinical assessments at baseline, 3‐month, 6‐month, and 12‐month intervals. Participants completed two picture description speech tasks at each timepoint. Clinical symptoms were assessed using the CDR, ADAS‐cog and MMSE. Biomarkers included neuroimaging markers (cortical thickness and volume) and CSF measurements of tau, p‐tau and AB40/42 ratio. Participants were grouped into terciles for each biomarker and group differences in speech characteristics were assessed. We also explored sensitivity to change by examining whether changes in speech were correlated with changes in biomarker values.

**Result:**

In the cross‐sectional analysis, several speech features showed significant group differences. The strongest associations were for cortical thickness, where average word length, frequency, use of nouns and pronoun:noun ratio showed stepwise differences across biomarker terciles. A similar, stepwise association with biomarker terciles was observed across all three clinical endpoints. Associations with CSF measures were notably weaker, with only 1 significant association (syntactic depth and p‐tau levels). Longitudinally, correlations between cortical thickness and speech characteristics were significant but weaker in magnitude (r<0.2) compared to correlations with clinical endpoints (r>0.4). One modest significant correlation was observed between speech and p‐tau (use of particles, r=‐0.17), while no significant correlations were observed between changes in p‐tau and changes in clinical endpoints.

**Conclusion:**

We found converging evidence for stepwise associations between neuroimaging markers and speech features previously shown to track clinical progression in AD. Associations with speech generally appeared weaker than associations with clinical endpoints, consistent with the different level of specificity of each measure (speech vs. general cognition and function). Results highlight a disease‐level association between speech parameters and neuroimaging markers of AD. Future work will help understand how these findings can be leveraged in screening and monitoring settings.

